# Supplementary material for: Distinct subtypes of proprioceptive dorsal root ganglion neurons regulate adaptive proprioception in mice
Source: Nat Commun. 2021 Feb 15;12:1026. doi: 10.1038/s41467-021-21173-9 (PMC7884389; doi:10.1038/s41467-021-21173-9)
Supplement: Supplementary file 1 — Supplementary Information [file 41467_2021_21173_MOESM1_ESM.pdf]

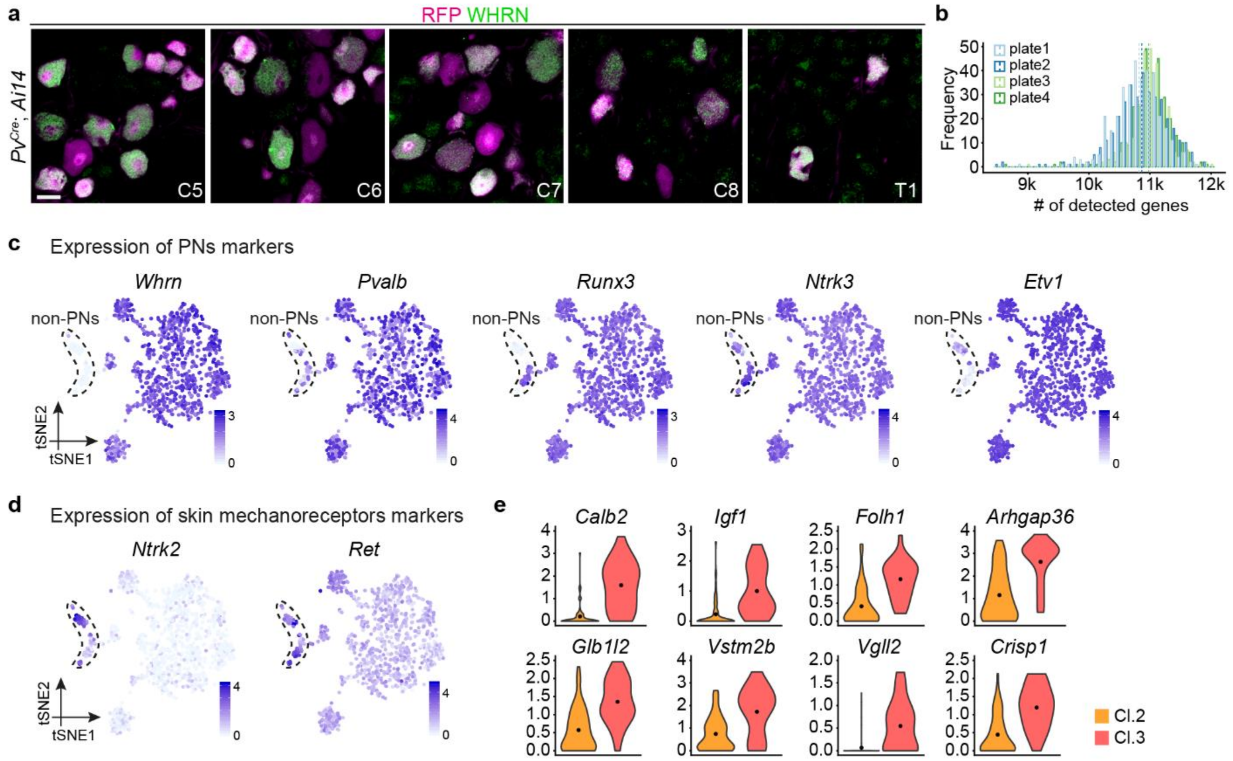

Supplementary Figure 1. Selection of PNs for scRNAseq analysis. a, Illustrative image of DRG sections of P40 PV^{Cre};*Ai14* mouse stained for WHRN, showing that the majority of RFP^{+} cells are PNs ($WHRN^{+}$) and visually all PNs are labeled by RFP. Scale bar: 20 µm. **b**, Histogram of number of detected genes per cell from 4 individual 384-well plates. A threshold is applied here at 7.5k genes to filter out low quality wells. **c**, tSNE showing the expression of canonical markers of PNs (*Whrn*, *Pvalb*, *Runx3*, *Ntrk3*, *Etv1*). **d**, tSNE showing the expression of canonical markers of mechanoreceptors (*Ntrk2*, *Ret*), those cells are removed from further analysis. **e**, Violin plots showing examples of differentially expressed genes in Cl.3 compared with Cl.2. Dots indicate the mean expression value.

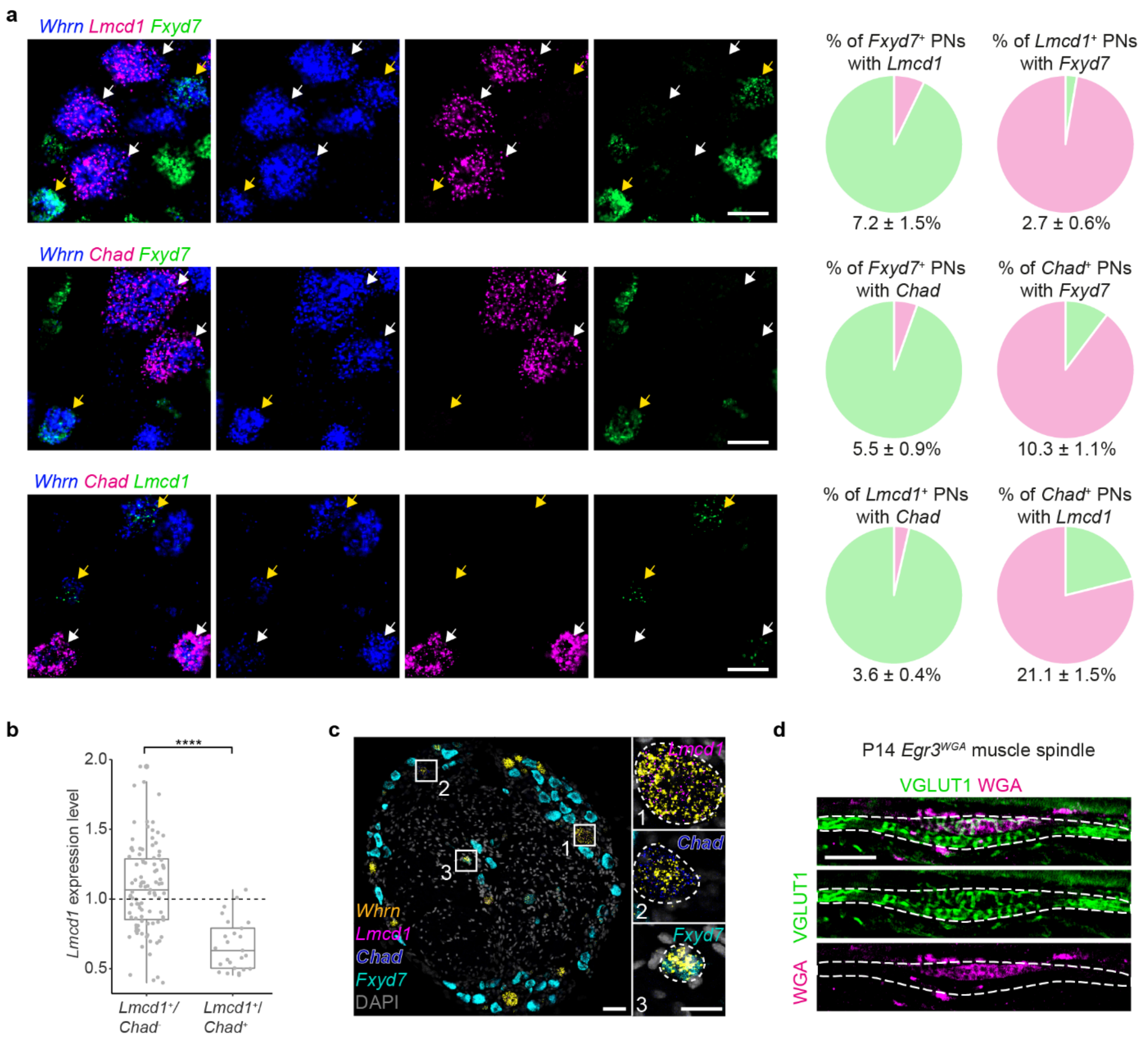

Supplementary Figure 2. Characterization of the major PN types. a, RNAscope showing combinatorial expression of markers of major PN types in P35 wildtype mice. Pie charts showing percentage overlap quantification (n = 3 animals). Scale bar: 20 µm. **b**, Quantification of *Lmcd1* expression in *Lmcd1*$^+$/*Chad*$^-$ and *Lmcd1*$^+$/*Chad*$^+$ PNs, normalized to the mean expression level of *Lmcd1* in individual section analyzed (n = 26 cells for *Lmcd1*$^+$/*Chad*$^+$ PNs, n = 124 cells for *Lmcd1*$^+$/*Chad*$^-$ PNs). Lower and upper hinges: first and third quartiles; the horizontal line: median; the whiskers extend to the value no further than 1.5 * IQR from the hinge; large dots: outliers. Two-tailed t-test, ****$p < 0.0001$. **c**, 4-plex RNAscope showing various soma sizes of the 3 major PNs populations (*Whrn*$^+$) identified by *Lmcd1*, *Chad* or *Fxyd7* expression on P54 DRG section. Scale bar: 50 µm. Scale bar of the micrograph: 20 µm. **d**, MSs of P14 *Egr3*^{WGA} mice stained for VGLUT1 and WGA to verify the expression site of WGA within MS. Scale bar: 50 µm. Source data are provided as a Source Data file.

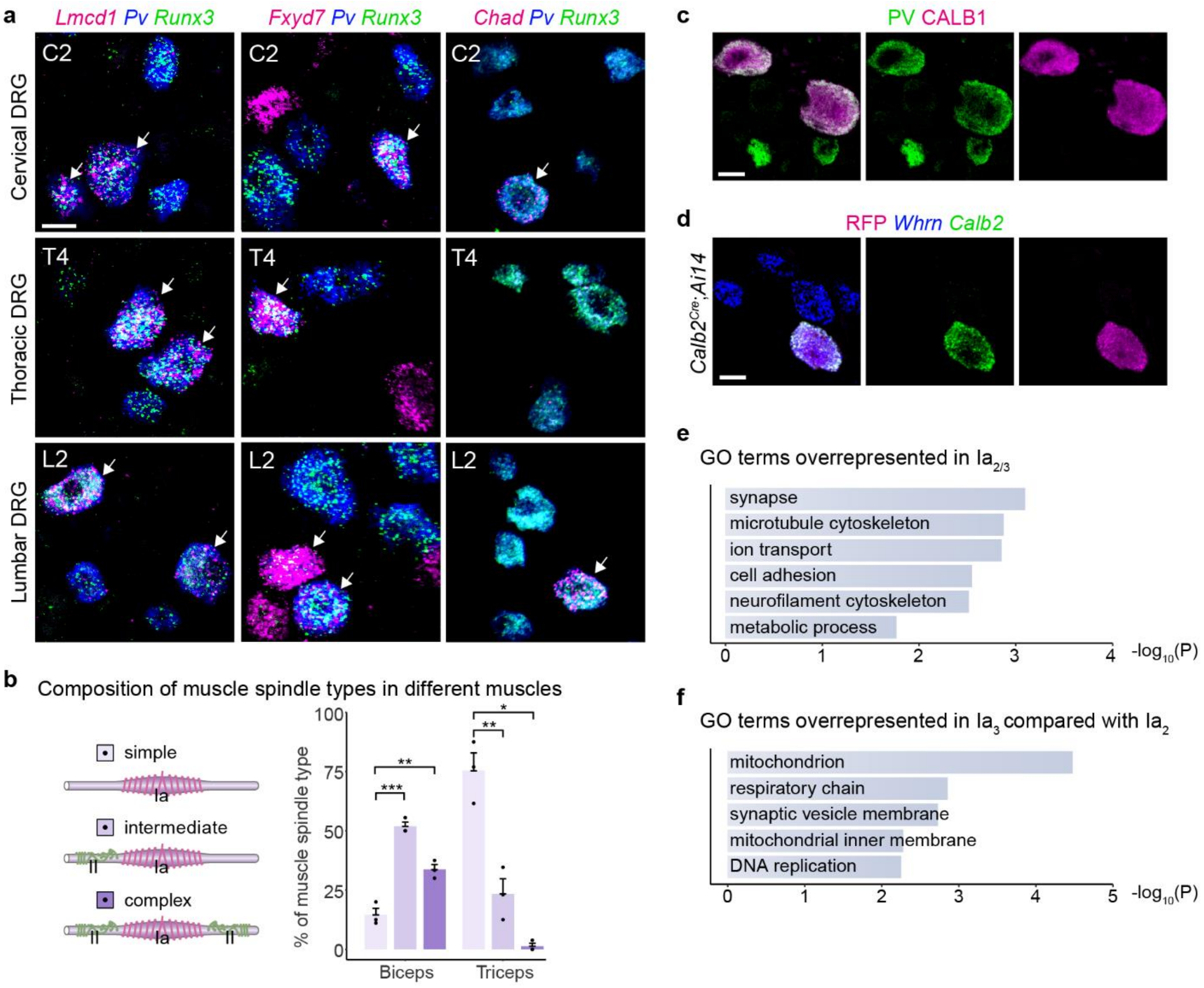

Supplementary Figure 3. Distribution and innervation of PNs types. a, Representative RNAscope images of the three major PNs types, labeled by identified markers (magenta), in individual DRG. PNs are labeled by *Pvalb*/*Runx3*. Scale bar: 20 µm. **b**, Proportion of different types of MSs in Biceps and Triceps quantified from MS stained for VGLUT1 (Ia- and II-PNs) and FXYD7 (II-PNs only). Data are presented as mean ± SEM (n = 3 animals); dots represent values from individual animals. Two tailed t-test, $*p < 0.05$, $**p < 0.01$, $***p < 0.001$. **c**, DRG section of P54 mice stained for CALB1 and PV, showing the larger soma sizes of CALB1$^+$ PNs (Ia$_{2/3}$-PNs). Scale bar: 20 µm. **d**, DRG section of P30 *Calb2^{Cre};Ai14* mice labeled for *Whrn* and *Calb2* using RNAscope, showing that all RFP$^+$ neurons are Ia$_3$-PNs (*Whrn*$^+$/*Calb2*$^+$). Scale bar: 20 µm. **e**, Gene Ontology (GO) analysis of differentially expressed genes in Ia$_2$- and Ia$_3$-PNs. **f**, GO analysis of differentially expressed genes in Ia$_3$-PNs compared with Ia$_2$-PNs. Source data are provided as a Source Data file.

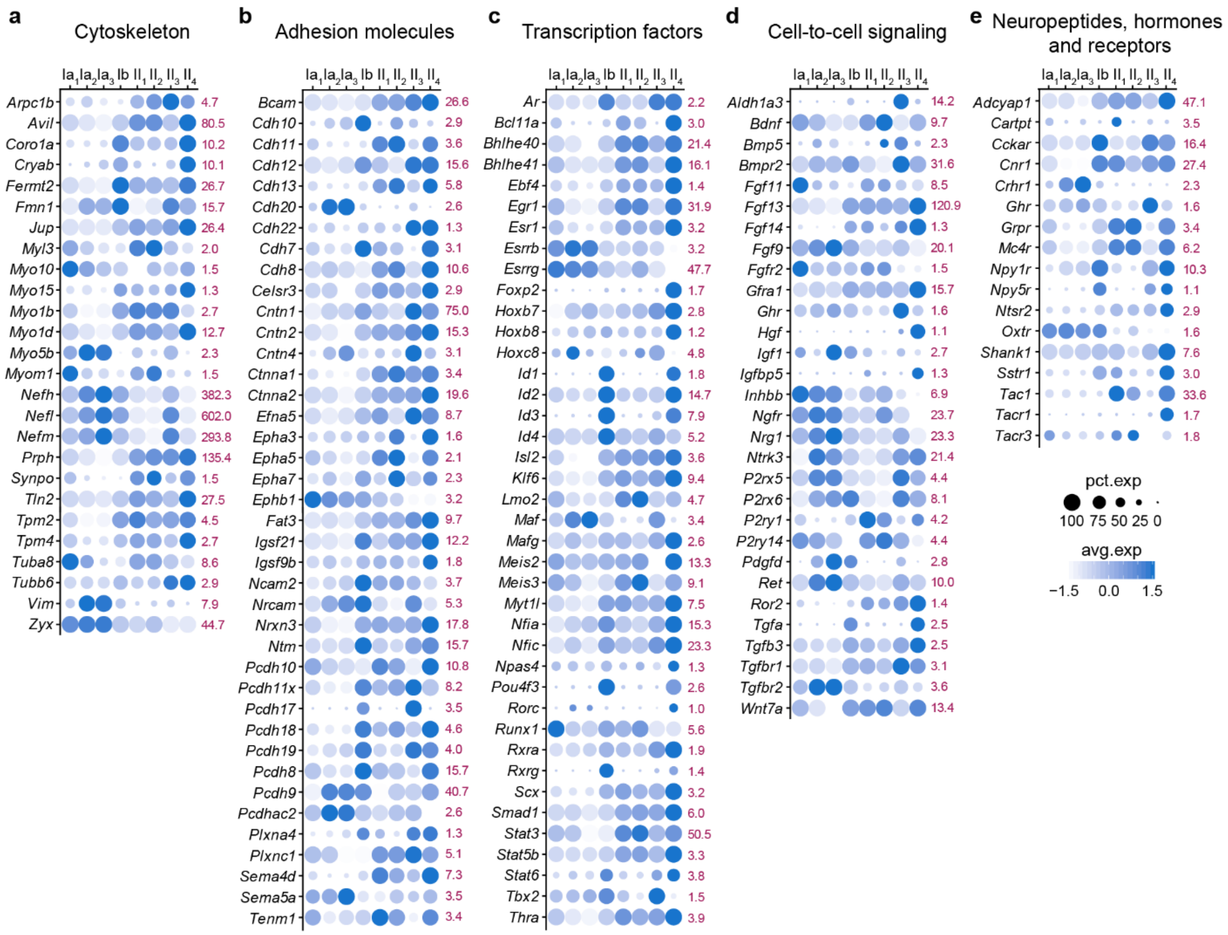

Supplementary Figure 4. Molecular delineation of PN types. a-e, Dot plots of differentially expressed genes among PN subtypes for general functional features, including “cytoskeleton”, “adhesion molecules”, “transcription factors”, “cell-to-cell signaling” and “neuropeptide, hormones, and receptors”. The highest mean of expression among the subtypes is specified in red.

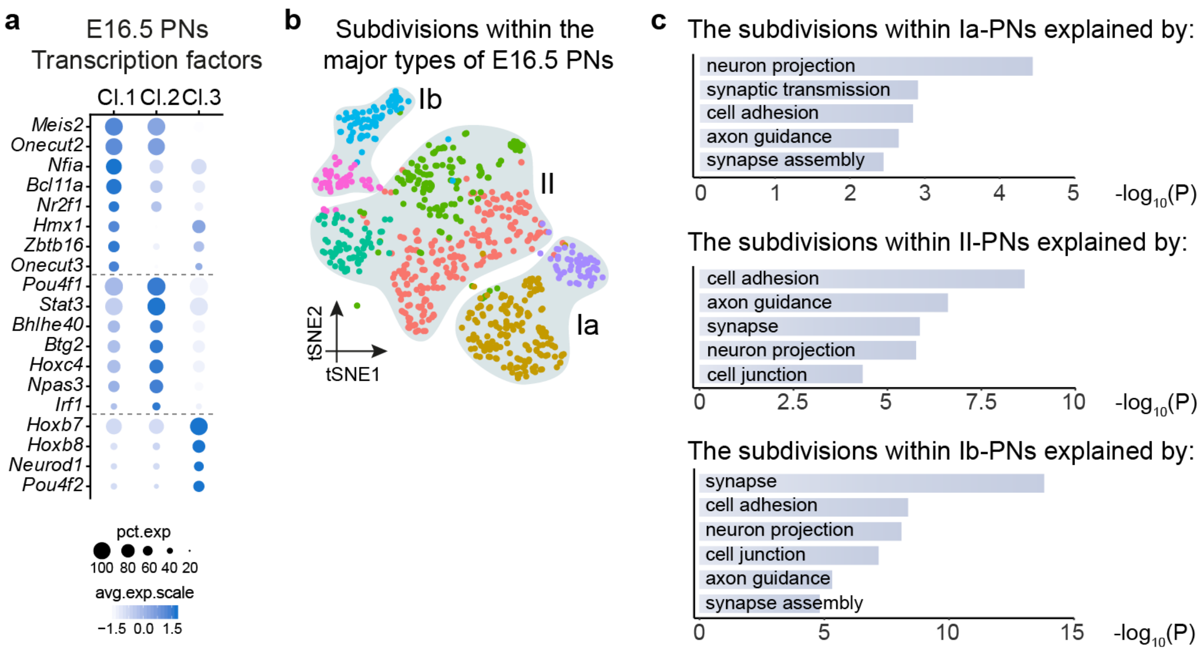

Supplementary Figure 5. PN subtypes at E16.5. a, Differentially expressed transcription factors in E16.5 clusters. **b**, tSNE showing molecularly distinct clusters within the three major types of PNs at E16.5. **c**, GO analysis of the differentially expressed genes among the clusters within each major type of PNs at E16.5.

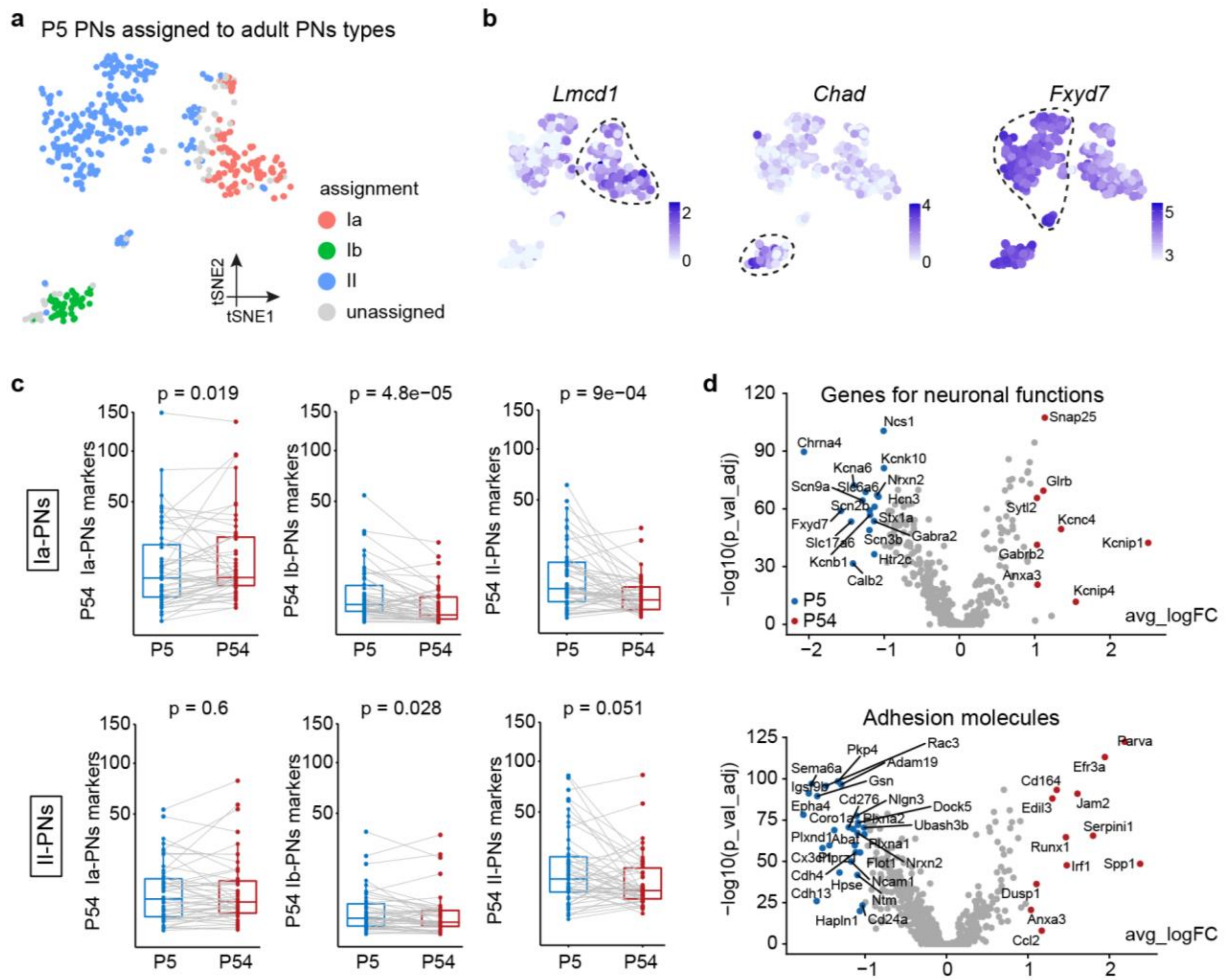

Supplementary Figure 6. Comparative analysis of gene expression in P5 and P54 PN types. **a**, tSNE of P5 PNs colored by assignment to major types of adult PNs using scmap. **b**, tSNE showing marker expression (*Lmcd1*, *Chad*, *Fxyd7*) of major PN types. **c**, Paired comparison of gene expression between P5 and P54 PNs types using top 50 markers of P54 major PNs types. Y axis are square root scaled. Lower and upper hinges: first and third quartiles; the horizontal line: median; the whiskers extend to the value no further than 1.5 * IQR from the hinge. Dots represent individual values. *P* values indicate the results of paired samples Wilcoxon test. **d**, Volcano plots of differentially expressed genes for neuronal functions or adhesion molecules between P5 and P54 type Ia PNs. Differentially expressed genes (absolute $\log_2$ fold change greater than 1 and corrected *p* value < 10^{-6}) are marked in blue for P5 and red for P54.

Supplementary Table 1. Primers used for genotyping of mouse strains

Mouse strain	Primer name	Sequence 5' to 3'	Primer type
PV^{Cre}	oIMR8290	CAGAGCAGGCATGGTGACTA	wt forward
PV^{Cre}	oIMR8291	AGTACCAAGCAGGCAGGAGA	wt reverse
PV^{Cre}	17283	AAATGCTTCTGTCCGTTTGC	mut forward
PV^{Cre}	oIMR9377	ATGTTTAGCTGGCCCAAATG	mut reverse
Ai14	oIMR9020	AAGGGAGCTGCAGTGGAGTA	wt forward
Ai14	oIMR9021	CCGAAAATCTGTGGGAAGTC	wt reverse
Ai14	oIMR9105	CTGTTCCTGTACGGCATGG	mut forward
Ai14	oIMR9103	GGCATTAAAGCAGCGTATCC	mut reverse
Egr3^{WGA}		AGAAAGATGATGAGCACCATGG	mut forward
Egr3^{WGA}		CTCTGGCAGCCTGCACCGCAAT	mut reverse
ChAT^{Cre}	oIMR6216	GTTTGCAGAAGCGGTGGG	wt forward
ChAT^{Cre}	oIMR6218	CCTTCTATCGCCTTCTTGACG	mut forward
ChAT^{Cre}	oIMR6217	AGATAGATAATGAGAGGCTC	common reverse
RGT		CACTTGCTCTCCCAAAGTCG	common
RGT		TAGTCTAACTCGCGACACTG	wt
RGT		GTTATGTAACGCGGAACTCC	mut
Calb1^{dgCre}		ACCAGGTTCGTTCACTCATGG	mut
Calb1^{dgCre}		AGGCTAAGTGCCTTCTCTACA	mut
Doc2b^{ddCre}		GGTGTAGCTGATGATCCGAATA	mut forward
Doc2b^{ddCre}		TGGGTTCAAATCCGCATCAG	mut reverse
