# Supplementary material for: Distinct subtypes of proprioceptive dorsal root ganglion neurons regulate adaptive proprioception in mice
Source: Nat Commun. 2021 Feb 15;12:1026. doi: 10.1038/s41467-021-21173-9 (PMC7884389; doi:10.1038/s41467-021-21173-9)
Supplement: Supplementary file 3 — Description of Additional Supplementary Files [file 41467_2021_21173_MOESM3_ESM.pdf]

Description of Additional Supplementary Files

Title: Supplementary Dataset 1.

Description: Genetic markers of adult PN subtypes. avg_logFC shows the log fold-change of the average expression of the cluster compared with all other clusters.

Title: Supplementary Dataset 2.

Description: Genetic markers specific to Ia3-PNs compared with Ia2-PNs. avg_logFC shows the log foldchange of the average expression of the Ia3-PNs compared with Ia2-PNs.
