# Supplementary material for: Distinct subtypes of proprioceptive dorsal root ganglion neurons regulate adaptive proprioception in mice
Source: Nat Commun. 2021 Feb 15;12:1026. doi: 10.1038/s41467-021-21173-9 (PMC7884389; doi:10.1038/s41467-021-21173-9)
Supplement: Supplementary file 6 — Reporting Summary [file 41467_2021_21173_MOESM6_ESM.pdf]

nature research

Corresponding author(s): Francois Lallemend

Last updated by author(s): Jan 5, 2021

Reporting Summary

Nature Research wishes to improve the reproducibility of the work that we publish. This form provides structure for consistency and transparency in reporting. For further information on Nature Research policies, see our Editorial Policies and the Editorial Policy Checklist.

Statistics

For all statistical analyses, confirm that the following items are present in the figure legend, table legend, main text, or Methods section.

n/a	Confirmed	
☐	☒	The exact sample size (*n*) for each experimental group/condition, given as a discrete number and unit of measurement
☐	☒	A statement on whether measurements were taken from distinct samples or whether the same sample was measured repeatedly
☐	☒	The statistical test(s) used AND whether they are one- or two-sided<br>*Only common tests should be described solely by name; describe more complex techniques in the Methods section.*
☒	☐	A description of all covariates tested
☐	☒	A description of any assumptions or corrections, such as tests of normality and adjustment for multiple comparisons
☐	☒	A full description of the statistical parameters including central tendency (e.g. means) or other basic estimates (e.g. regression coefficient) AND variation (e.g. standard deviation) or associated estimates of uncertainty (e.g. confidence intervals)
☐	☒	For null hypothesis testing, the test statistic (e.g. *F*, *t*, *r*) with confidence intervals, effect sizes, degrees of freedom and *P* value noted<br>*Give P values as exact values whenever suitable.*
☒	☐	For Bayesian analysis, information on the choice of priors and Markov chain Monte Carlo settings
☒	☐	For hierarchical and complex designs, identification of the appropriate level for tests and full reporting of outcomes
☒	☐	Estimates of effect sizes (e.g. Cohen's *d*, Pearson's *r*), indicating how they were calculated

Our web collection on statistics for biologists contains articles on many of the points above.

Software and code

Policy information about availability of computer code

Data collection	Smart-Seq2 protocol was performed on single isolated cells in the lab (for the rabies virus traced PNs) or by Eukaryotic Single Cell Genomics Facility at SciLifeLab, Stockholm (for the E16.5, P5 and P54 PNs). The data pre-processing (demultiplexing, annotation) was done by Eukaryotic Single Cell Genomics Facility with the following steps:<br>-The samples were analyzed by first demultiplexing the fastq files using deindexer (http://github.com/ws6/deindexer) using the nextera index adapters and the 384 well layout.<br>-Individual fastq files were then mapped to the relevant genome assembly using the STAR aligner (Dobin et al. Bioinformatics 2013) using 2-pass alignment to have improved performance of de novo splice junction reads, filtered for only uniquely mapping reads that are saved in the BAM file.<br>-The expression values were computed per gene as described in Ramsköld et al. (2009 PLoS Comp Biol), using uniquely aligned reads and correcting for the uniquely alignable positions using MULTo (Storvall et al. 2013 PLoS ONE).
Data analysis	Most of the downstream analysis of scRNAseq data of E16.5, P5 and P54 PNs followed the same pipeline using R package Seurat (version 2.4). Projection of scRNAseq data across data sets was done using R package scmap (version 1.10). Statistical data analysis was performed with Microsoft Excel. More details are presented in the Methods section.

For manuscripts utilizing custom algorithms or software that are central to the research but not yet described in published literature, software must be made available to editors and reviewers. We strongly encourage code deposition in a community repository (e.g. GitHub). See the Nature Research guidelines for submitting code & software for further information.

Data

Policy information about availability of data

All manuscripts must include a data availability statement. This statement should provide the following information, where applicable:

- Accession codes, unique identifiers, or web links for publicly available datasets
- A list of figures that have associated raw data
- A description of any restrictions on data availability

All data is available in the main text or the supplementary materials, apart from the scRNAseq transcriptomic data which is accessible at GEO data repository (accession code: GSE156180): https://www.ncbi.nlm.nih.gov/geo/query/acc.cgi?acc=GSE156180.

Field-specific reporting

Please select the one below that is the best fit for your research. If you are not sure, read the appropriate sections before making your selection.

☒ Life sciences ☐ Behavioural & social sciences ☐ Ecological, evolutionary & environmental sciences

For a reference copy of the document with all sections, see nature.com/documents/nr-reporting-summary-flat.pdf

Life sciences study design

All studies must disclose on these points even when the disclosure is negative.

Sample size	For the scRNAseq data analysis, 809, 479, 1109 single cells were included for E16.5, P5, P54 PNs, respectively. The sample size was estimated based on the assumed number of cell types, minimal fraction of rarest cell type and minimal desired cells per type, using the web application by Satija lab (https://satijalab.org/howmanycells). For other experiments, n>=3 animals.
Data exclusions	For the scRNAseq data analysis, the exclusion criteria varied between different plates and different stages, more details is provided in the Methods section. In general, based on the distribution of number of detected genes in the cells, cells with very few detected genes (Low-quality cells or empty wells) and aberrantly high detected genes (doublets or multiplets) were removed from the downstream analysis. For the analysis of P5 and P54 data, glial contaminated neurons were removed based on glial markers expression.
Replication	For scRNAseq experiments, replicates were done for all stages: E16.5: 3 plates; P5: 2 plates; P54: 4 plates. For immunostaining and RNAscope experiments, replicates were performed in often n>=3 animals even in cases where only representative images were presented in the figure.
Randomization	No randomization was performed since no experimental groups were used.
Blinding	For majority of the experiments in this study, only wild-type animals (not exposed to any experimental conditions) were used, thus not applicable to blinding. For the experiments comparing different groups, e.g. voluntary running vs. sedentary mice in Fig. 6, standardized procedure was applied to both groups.

Reporting for specific materials, systems and methods

We require information from authors about some types of materials, experimental systems and methods used in many studies. Here, indicate whether each material, system or method listed is relevant to your study. If you are not sure if a list item applies to your research, read the appropriate section before selecting a response.

Materials & experimental systems

n/a	Involved in the study	
☐	☒	Antibodies
☒	☐	Eukaryotic cell lines
☒	☐	Palaeontology and archaeology
☐	☒	Animals and other organisms
☒	☐	Human research participants
☒	☐	Clinical data
☒	☐	Dual use research of concern

Methods

n/a	Involved in the study	
☒	☐	ChIP-seq
☒	☐	Flow cytometry
☒	☐	MRI-based neuroimaging

Antibodies

Antibodies used	Primary antibodies used were: Rabbit anti-WHRN (from Joriene de Nooij lab; de Nooij et al, 2015), mouse anti-ISLET1 (DSHB, cat#39.4D5), chicken anti-RFP (Rockland, cat#600-901-379S), rabbit anti-VGLUT1 (SYSY, cat#135303), guinea pig anti-VGLUT1 (SYSY, cat#135304), rabbit anti-FXYD7 (Sigma-Aldrich, cat#HPA026916), rabbit anti-LMCD1 (Human Protein Atlas, cat#HPA024059), goat anti-WGA (Vector Laboratories, cat#AS-2024), mouse anti-BRN3C (Santa Cruz Biotechnology, cat#sc2881980), goat anti-PV (Swant, cat#PVG-213), rabbit anti-PV (Swant, cat#PV27), rabbit anti-CART (from Igor Adameyko lab; Phoenix Pharma., cat#H-003-62), rabbit

	anti-RUNX1 (from Thomas Jessell lab; Chen et al., 2006), rabbit anti-CALB1 (Swant, cat#CB-38a), goat anti-CHAT (Millipore, cat#AB144p), DAPI (Invitrogen, cat#D1306)<br>Secondary antibodies used were: Anti-chicken cy3 (Jackson, cat#703-165-155), anti-rabbit 488 (Life Technologies, cat#A-21206), anti-goat 647 (Life Technologies, cat#A-21447), anti-rabbit 555 (Life Technologies, cat#A-31572), anti-goat 488 (Life Technologies, cat#A-11055), anti-rabbit 647 (Life Technologies, cat#A-31573), anti-mouse 647 (Life Technologies, cat#A-31571)
Validation	All antibody validations are available on manufacturers' websites or previous published studies, Wang/Wu et al., 2019, de Nooij et al., 2015, Chen et al., 2006.

Animals and other organisms

Policy information about studies involving animals; ARRIVE guidelines recommended for reporting animal research

Laboratory animals	Mice were housed in cages in groups, with food and water ad libitum, under 12h light-dark cycle conditions. PVCre;Ai14 mice were crossed from PVCre (from The Jackson Laboratory (Hippenmeyer et al., 2005)) and Ai14 (from The Jackson Laboratory (Madisen et al., 2010)), and used to genetically label PNs for scRNAseq experiments. Egr3WGA mice (de Nooij et al., 2013) were used to specifically label MSs-innervating PNs. ChATCre;RGT mice were crossed from ChATCre (from Ole Kiehn lab (Rossi et al., 2011)) and RGT (from The Jackson Laboratory (Takatoh et al., 2013)), and used to specifically express avian receptor protein TVA and rabies glycoprotein in MNs for rabies virus infection. Calb2Cre;Ai14 mice (Taniguchi et al., 2011) were used to genetically label Calb2+ cells in DRG for neuroanatomical tracing. Calb1dgCre;Ai14 mice were crossed from Calb1dgCre (from The Jackson Laboratory (Daigle et al., 2018)) and Ai14, and used to genetically label and trace the nerve endings of adult Calb1+ PNs. Doc2bdgCre;Ai14 mice were crossed from Doc2bdgCre (generated in collaboration with Gurumurthy lab) and Ai14, and used for lineage tracing of embryonic Doc2b+ PNs. C57BL/6J mice were received from The Jackson Laboratory (stock #000664) and used for most experiments unless otherwise specified. Animals of either sex were included in this study.
Wild animals	No wild animals were used in the study.
Field-collected samples	No field collected samples were used in the study.
Ethics oversight	All animal experiments were approved by the local ethical committee (Stockholms Norradjurförsöksetiska nämnd, Sweden) and conducted following the ethical guidelines described in the Swedish Animal Agency's Provisions and Guidelines for Animals Experimentation Recommendations.

Note that full information on the approval of the study protocol must also be provided in the manuscript.
